# Parameter Mapping Sonification of Human Olfactory Thresholds

**DOI:** 10.3390/biology12050670

**Published:** 2023-04-28

**Authors:** Jean-Luc Boevé, Rudi Giot

**Affiliations:** 1 OD Taxonomy and Phylogeny, Royal Belgian Institute of Natural Sciences, Rue Vautier 29, 1000 Brussels, Belgium; 2 Research Laboratory in the Field of Arts and Sciences, Institut Supérieur Industriel de Bruxelles, Rue Royale 150, 1000 Brussels, Belgium

**Keywords:** human olfactory detection, sonification, parameter mapping, random setting, chemical parameter, sound parameter

## Abstract

**Simple Summary:**

It is challenging to deduce the bioactivity of volatile compounds from their chemical characteristics. We therefore previously used parameter mapping sonification to study volatiles secreted by some insects that repel predators. Chemical parameters from single volatiles were linked to sound parameters. The peak sound pressure values from the gathered audio clips contain information about the repellent effect of the compounds. Here, human olfactory thresholds were investigated. The volatiles were subjected to parameter mapping, and the results show that these thresholds are correlated with the peak sound pressures. More generally, the results illustrate that the sonification of volatiles helps to better understand their bioactivity.

**Abstract:**

An objective of chemical ecology is to understand the chemical diversity across and within species, as well as the bioactivity of chemical compounds. We previously studied defensive volatiles from phytophagous insects that were subjected to parameter mapping sonification. The created sounds contained information about the repellent bioactivity of the volatiles, such as the repellence from the volatiles themselves when tested against live predators. Here, we applied a similar sonification process to data about human olfactory thresholds. Randomized mapping conditions were used and a peak sound pressure, Lpeak, was calculated from each audio file. The results indicate that Lpeak values were significantly correlated with the olfactory threshold values (e.g., r_S_ = 0.72, t = 10.19, *p* < 0.001, Spearman rank-order correlation; standardized olfactory thresholds of 100 volatiles). Furthermore, multiple linear regressions used the olfactory threshold as a dependent variable. The regressions revealed that the molecular weight, the number of carbon and oxygen atoms, as well as the functional groups aldehyde, acid, and (remaining) double bond were significant determinants of the bioactivity, while the functional groups ester, ketone, and alcohol were not. We conclude that the presented sonification methodology that converts chemicals into sound data allows for the study of their bioactivities by integrating compound characteristics that are easily accessible.

## 1. Introduction

Living organisms produce a huge diversity of chemical compounds that are often used in defence against attacking predators. Defensive compounds constitute interspecific chemical signals that are adaptively beneficial to the emitter but not the receiver [1]. Chemical compounds with a defensive function occur in many plants and animals [2,3,4]. The high diversity of defensive chemicals throughout living organisms is manifest among different species, but also intra-specifically, as a species often emits a complex mixture of defensive compounds [5].

**Figure 1:**
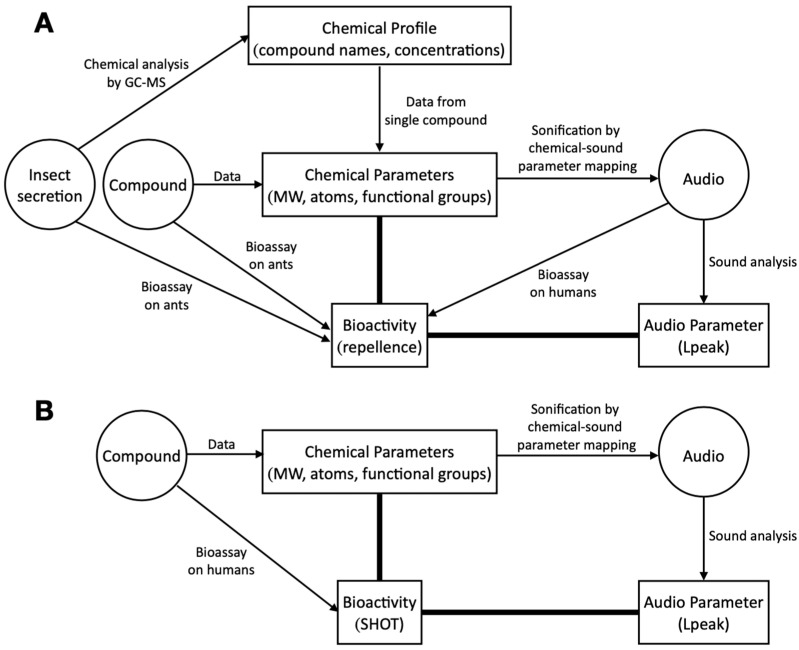
Schematic representations of the causal relationships and correlations between elements dealing with volatile compounds studied by sonification in Boevé and Giot [12] (**A**) and in this study (**B**). In (**B**), data about bioassay on humans were gathered from the literature. Datasets are mentioned in a box, ‘objects’ in a circle. (**A**) causal relationship is shown by an arrow, and a statistical correlation is shown by a thick bar. Notice that in (**A**), the insect secretion was tested on ants indirectly by confronting them with live insects. Gaz chromatography-mass spectrometry (GC-MS). Molecular weight (MW). Standardized human olfactory threshold (SHOT). Peak sound pressure (Lpeak). For more information, see text.

Volatiles acting as repellents, that is, at distance and through the olfactory sense [6], are often used by phytophagous insects against the attack of predators [1,7,8,9]. As a first application of sonification [10,11] to chemical ecology, several insect species belonging to a group of phytophagous hymenopterans, the nematines, were studied [12]. From the chemical profile of the defensive volatile secretions of these insects, each single compound, or molecule, was subjected to sonification, by which chemical parameters were converted into sound parameters (Figure 1). The single compound audio obtained in this way were combined into new, species-specific audio based on the relative concentration of each compound in the given species (not shown in Figure 1). The repellent effect of the single or mixed compounds was tested by bioassays on predatory ants, while both sets of corresponding audio were tested on humans. As a measure of loudness, or volume, the maximum peak reached by the sound pressure (Lpeak, in dB) was calculated from each audio. Testing correlations between Lpeak values and bioassay results revealed that both datasets are significantly positively correlated, which suggests that the audio clips contain information about the repellent bioactivity (Figure 1). Thus, practically speaking, testing audio on humans can be replaced by the more convenient process of measuring Lpeak values from the audio [12].

Any sonification process involves some subjectivity and arbitrary choices when assigning datasets to sound parameters. This is illustrated in many studies applying sonification to various scientific, technical, and societal domains: chemistry [13,14], genomics–proteomics–medicine [15,16,17,18,19,20,21,22,23], animal migration [24], geographic data [25], climatic data [26], seismology [27,28], petroleum engineering [29], astronomy [30], mathematics [31], internet monitoring [32], market data [33], and vision impairment [34,35]. Those studies about (organic) chemistry show that sonification allows an efficient analysis of experimental data and a pattern recognition of chemical samples [13,14,18,20,22,23]. In neuroscience and cardiology, it allows a better monitoring of physiological activities than by statistical approaches [16,21].

Here, one aim was to better understand the pros and cons of the sonification methodology by studying a biological system other than insect predator–prey relationships. Bioassays of volatiles rarely involve numerous compounds tested in identical conditions on one species, an exception being volatiles tested on humans [36,37,38]. Our aim is to propose an alternative approach to the use of regression models to estimate the bioactivity of volatiles on humans [39,40,41,42,43,44,45,46,47]. These models involve variables such as the presence/absence of functional groups, such as aldehyde or acid, but also other variables that may be difficult to obtain from the literature on compounds rarely encountered in living organisms. More recent data modelling includes two-dimensional gas chromatography retention parameters [48] to determine pungency or irritation thresholds [43,44,49,50]. Here, the human olfactory thresholds of single molecules from Devos et al. [41] were compared with the Lpeak values obtained by the sonification of the same molecules (Figure 1). We reduced the degree of subjectivity by randomizing the mapping conditions. Furthermore, we tested sound parameters separately to evaluate their specific impact on the audio. By randomizing and gradually refining the mapping conditions, we succeeded in increasing the correlation strength between olfactory thresholds and Lpeak values.

## 2. Materials and Methods

### 2.1. Human Olfactory Threshold

As a bioactivity measure, the standardized human olfactory threshold (SHOT, in a log unit of ‘olfactory power’ that can be converted into ppm or ppb units) was acquired from a dataset of 529 molecules [41] based on more than 100 literature references. It includes for each molecule the following data: molecule name, synonyms, reference number, chemical formula, molecular weight (MW), and two SHOT values, viz. mass (d1) or volume (d2) weighted. In short, the higher a SHOT value, the easier the volatile is detected by the human nose. In the present study, the following additional data were gathered from PubChem [51] and other online sources [52]: the Chemical Abstracts Service (CAS) registry number to resolve synonymy ambiguities of chemical names compared with those from its own data sources, and the functional groups. For statistical tests not directly related to the sonification, a first subset of 272 molecules was selected (Table S1). These molecules were mainly aliphatics, and they contained only carbon, hydrogen, and oxygen for some of them. For the sonification, a more restricted subset of 100 molecules was used, being listed in Devos et al. [41] and Abraham et al. [40]. The latter reference is based on Nagata [53] and others, who measured an odour detection threshold (ODT, in ppm, *v*/*v*, and negatively correlated with the SHOT) from which Abraham et al. [40] derived values of log (1/ODT).

### 2.2. Sonification

**Figure 2:**
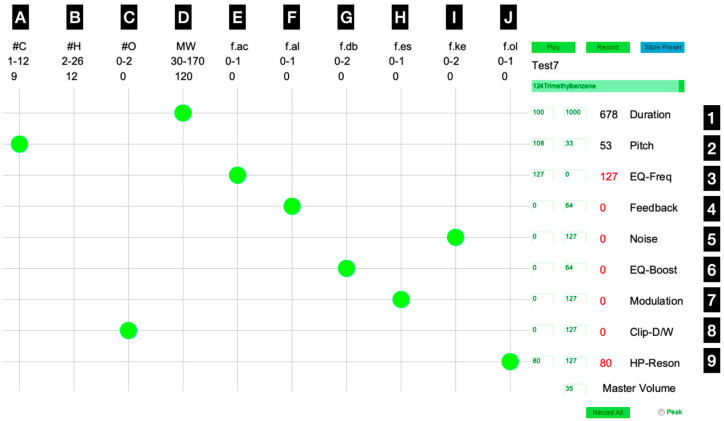
Screenshot of the layout interface used in an illustrative condition of parameter mapping by which chemical parameters are linked to sound parameters (green nodes). Letters and digits with a black background are added to the screenshot, as the mapping preset names refer to them in Table 1. By clicking the “Record All” (below, right), the Processing script performs a batch export of one audio file per molecule listed in a CSV file. For more information, see text.

Working under MacOS Monterey (version 12), the sonification process was applied on single molecules. The chemical descriptors (Table S1) were linearly scaled to fit the general musical instrument digital interface (MIDI) specification, and subjected to a process of parameter mapping [11,25] by using the synthesizer Massive version 1.5.1 (R637) (Native Instruments, Berlin, Germany) and an application written in Processing version 2.2.1 (Processing Foundation, MIT Media Laboratory, Cambridge, MA, USA) (Figure 2).

A single synthesizer’s preset sound was assigned to all molecules; it is close to the preset sound “Cloud N9” available in Massive. Only two chemical–sound links (i.e., nodes) were left unchanged throughout the study: a negative relationship was set between the number of carbon atoms in the molecule (1–12; ‘#C’) and the note pitch (MIDI note range of 108–33; see ‘Pitch’ in Figure 2) of the sound assigned to it; a positive relationship was set between the MW (30–170) of the molecule and the note duration (1–10 s; ‘Duration’) of the sound assigned to it. These two links were kept the same to reflect that smaller, compared with larger, molecules are more volatile, thus evaporating more rapidly, which we also linked to the perception of higher sound frequencies. The other chemical and sound parameters were linked together in various combinations across 50 settings to assess the statistical correlation of the resulting audio Lpeak values with the SHOT values (see later). The chemical parameters were the number of oxygen atoms (0–2; ‘#O’), acid groups (0–1; ‘f.ac’), aldehydes (0–1; ‘f.al’), double bonds (0–2; ‘f.db’; not part of other functional groups), esters (0–1; ‘f.es’), ketones (0–2; ‘f.ke’), and alcohols (0–1; ‘f.ol’). The sound parameters were the equalizer frequency (MIDI control 127–0, i.e., decreasing; ‘EQ-Freq’), feedback amplitude (0–127; ‘Feedback’), noise metallic amplitude (0–127; ‘Noise’), equalizer boost (0–64; ‘EQ-Boost’), modulation oscillator filter FM (0–127; ‘Modulation’), insert 1 hardclipper dry/wet (0–127; ‘Clip-DW’), and filter 2 high-pass 4 resonance (80–127; ‘HP-Reson’).

The recorded WAV sounds were gathered via the virtual audio driver BlackHole 16ch version 1.3.0.65 [54], a single batch record of 100 molecules lasting 40–50 min. Different mapping conditions were referred to by a code corresponding to the node positions, with letters “A” to “J” for the chemical parameters and numbers “1” to “9” for the sound parameters (Figure 2). For instance, A2-D1--C8-E3-F4-G6-H7-I5-J9 represents the mapping condition shown in Figure 2. Notice that the number of hydrogen atoms (“B”) was never used, since it is obviously correlated with the #C in organic compounds. As mentioned, we always used the two same nodes that connect the #C with pitch (A2), and the MW with duration (D1). For the seven other chemical parameters (“C”, “E”, “F”, “G”, “H”, “I”, and “J”), 24 assignments to sound parameters “3” to “9” were randomly selected for testing out of 5040 possible combinations; the assignments were randomized by using random functions in Excel. More specific mapping conditions were also tested.

A general workflow was applied as follows: Two CSV files contained the compound name and its chemical descriptors, one file for the 272 molecules, the other for the subset of 100 molecules (Table S1). The master volume of Massive was kept the same, the wheel cursor being positioned as it would be at 10:30 on an analogue clock. In the Processing script, “silence threshold” was set at −60 dB, “duration” at 5 s, and in the mapping interface, the volume was set at 35. A batch record of all 100 molecules led to as many WAV files. Each audio file was analyzed with another application written in Processing to calculate the Lpeak values that were then averaged over the left and right audio channels [12,55]. Overall, at least 5000 audio files were generated during the study.

To estimate the respective impact of each sound parameter on Lpeak, each one was tested separately. To this end, a CSV file contained five theoretical molecules with the following #C vs. MW values: 1 vs. 30, 3 vs. 65, 7 vs. 100, 10 vs. 135, and 12 vs. 170, respectively. The #C was mapped to ‘Pitch’ and MW to ‘Duration’. Audio was gathered with this simple mapping (“A2-D1”, see above) and then with a supplementary node that successively linked a third chemical parameter with each of the seven remaining sound parameters.

### 2.3. Statistical Analyses

Spearman rank-order correlations were tested and computed online [56] between the SHOT d1 values and the Lpeak values originating from the batch sonification of 100 molecules. The calculation was performed for each mapping condition. These association analyses were explorative in nature and used the same SHOT values throughout the correlation tests. The correlations were followed by the Holm’s sequential Bonferroni correction at significance level α = 0.05. Such Spearman correlations corrected for multiple testing were also performed between SHOT d1 vs. d2, d1 vs. Lpeak, and log (1/ODT) vs. Lpeak.

To assess the added value of the sonification process, the relationships among the chemical parameters, or variables, were modelled by multiple linear regressions (MLRs) using PAST version 4.09 [57]. SHOT d1 was considered as the dependent variable, the eight independent variables being the number of carbon atoms, oxygen atoms, and the functional groups: acid, aldehyde, ester, ketone, alcohol, and (remaining) double bond.

## 3. Results

**Figure 3:**
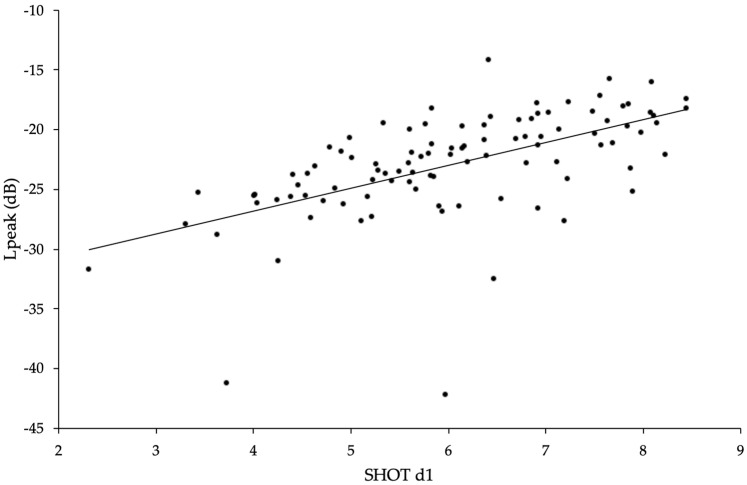
Scatterplot and regression line of the SHOT d1 versus Lpeak values for 100 molecules. The molecules and their values are listed in Table S1. The regression formula is Y = −34.474 + 1.9131 X.

Among 24 sonification batch runs selected randomly (except for the nodes “A2” and “D1”), 20 were significantly correlated with the SHOT d1 values, and five resulted in a Spearman’s correlation coefficient (r_S_) > 0.45 (Table 1, set01 to set24). In these five mapping conditions, the node “E3” occurred five times and “F4” three times. Therefore, 24 new conditions were selected randomly out of 120 possible combinations by keeping constant the nodes “A2”, “D1”, “E3”, and “F4” (Table 1, set26 to set30 plus set54 to set72). The 24 consequent correlations were all significant (*p* < 0.001, Spearman rank-order correlation; Table 1). A detailed survey of these 24 batches of 100 molecules revealed that Lpeak values tended to be equal or close to 0 dB for molecules containing an aldehyde. This suggested that the sound parameter Feedback, to which this function group was assigned, had a strong impact on the Lpeak values. By restricting the MIDI range of Feedback from 0 to 64 (instead of 0–127), all Lpeak data points had less than −10 dB (Figure 3), although the r_S_ was not increased (see set57b and set73, Table 1). At the lowest dB values, two outliers were observed with less than −40 dB, which corresponded to the data point of methanol and methanal (Figure 3).

Significant correlations were obtained by comparing the bioactivity variables SHOT d1 with d2, SHOT d1 with Lpeak, and log (1/ODT) with Lpeak (Table 2).

Based on the list of 272 molecules, an MLR using SHOT as a dependent variable led to an overall significant regression (R^2^ = 0.43, F_8.263_ = 25.19, and *p* < 0.0001), yet excluding the independent variables ester, ketone, and alcohol (Table S2). In a following MLR, these three functional groups were discarded. Such a reduced model, containing only the number of carbon and oxygen atoms, as well as the functional groups acid, aldehyde, and double bond, still provided a significantly better fit than the null model (R^2^ = 0.42, F_5.266_ = 38.39, and *p* < 0.0001). Based on the subset of 100 molecules, a third MLR using these same dependent and five independent variables also resulted in a significant better fit than the null model (R^2^ = 0.54, F_5.94_ = 21.91, *p* < 0.0001) without excluding any of the independent variables (Table S2).

**Figure 4:**
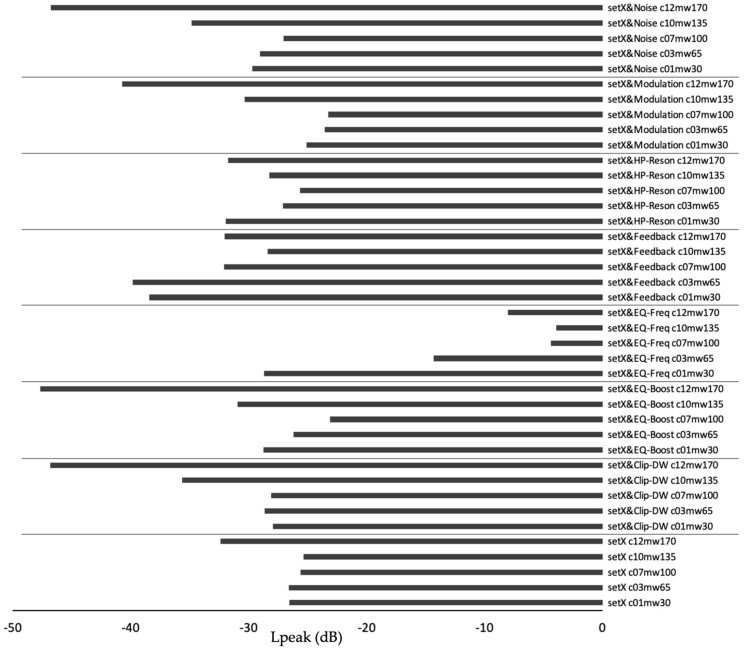
Peak sound pressure obtained by separately testing sound parameters. Five theoretical, small to large, molecules were created in a CSV file (named “setX”). They were used in parameter mapping conditions that included a reduced set of linking nodes. For more explanation, see text.

Testing the respective impact of each sound parameter on Lpeak revealed that Lpeak values were generally lower (<−30 dB) for the largest molecule compared with the smaller ones (Figure 4). The sound parameter ‘EQ-Freq’ was particular in that larger molecules led to high Lpeak values over −10 dB (Figure 4).

## 4. Discussion

Starting with a dataset about volatile compounds, a sonification process was performed by randomly selecting the parameter mapping conditions. Correlations were calculated between the values of Lpeak from the audio clips and the thresholds, SHOTs, at which humans perceive these volatiles. Relationships between chemical and bioactivity variables were already studied by other methodologies, but acquiring these variables often requires a bioassay series, advanced computing, and/or analytical chemistry [40,48,58]. In the present study, the highest achieved Spearman correlation coefficients (of 0.7) can be interpreted as moderate to nearly strong [59], and outliers were observed. However, interestingly, the overall (i.e., multiple) correlation coefficients from the MLRs reached a similar relationship strength, and the datapoint distribution on the scatterplot indicated no polynomiality. Thus, for the chemical variables considered here, an equivalent performance was reached by using Lpeak values resulting from the sonification process versus using classical statistics only, yet it should be possible to still enhance the relationship strength by further adapting the parameter mapping conditions. This way, and compared with the linear modelling approach, parameter mapping sonification would provide a better insight into the influence of chemical attributes on a bioactivity.

In chemical ecology, bioassays are used to obtain quantitative data about the effect of volatiles on organisms. For instance, rates of repellence are gathered from ants confronted to volatile vapours [1,8,9,60], while the SHOT values used here were gathered from humans detecting odours [53]. Screening volatiles by such experiments allows the ranking of their activity level. In this context, sonification is a complementary or alternative way to obtain comparative, quantitative data. Its advantage is that bioassays are less necessary; such experiments using living organisms may be difficult to set up [12]. More generally, the sonification of medical measures, biological and seismological data, etc., sometimes allows a better interpretation of raw data than via their visual representation that may be less informative [14,20], but listening to such sonified data often requires a preliminary auditory training of the operator [13,14,21]. A training is unwarranted for humans used in bioassays to test their response against volatiles, since they are “detectors” that react upon perceiving a chemical (or an auditory) stimulus, not “translators” that should interpretate the meaning of raw data obtained from analytical chemistry, physiology, or physics.

Reference [12] made three levels of arbitrary choices: (1) the assignment in Massive of a specific preset sound to each chemical class; (2) a general, preselected setting of the parameter mapping condition, on which most of the study relies, then randomizing the conditions to find those leading to higher correlation results; and (3) the mixing of molecule audio into a species audio, according to the relative chemical concentrations in the original insect secretion. Here, the first and third levels were not considered, the first one since the present sound was kept the same for all sonified molecules, and the third one because only single molecules were studied.

From the list of molecules analyzed by parameter mapping, the molecule with lowest #C was assigned to the sound with the highest frequency, and vice versa. In Boevé and Giot [12], the molecules had a #C from 2 to 29 and a MW from 32 to 425, while in the present study, these ranges were 1–12 and 30–170, respectively. Generally, any list of molecules may lead to a particular scaling between chemical and sound parameters, possibly causing the audio of identical molecules from different lists to sound at differing frequencies.

Results from the MLR statistics reveal that among the six functional groups, acid, aldehyde, double bond, ester, ketone, and alcohol, the last three ones can be considered as dispensable, although all six were used in the Spearman correlations between Lpeak versus SHOT and log (1/ODT). Furthermore, the MW of organic molecules is obviously correlated with their #C. Therefore, only the second variable was used in MLRs, while both variables were kept in the sonification. This is because we consider parameter mapping as resulting in audio characteristics that are not properly inferable from a particular chemical parameter. In fact, each distinct sound parameter influenced Lpeak differently, and in a variable way depending on sound frequency (determined by the MW). In contrast to sound frequency (pitch) and duration, the Lpeak, as a measure of sound loudness, was not directly mapped with a chemical trait, although ‘EQ-Freq’ taken alone increased the Lpeak values. The sound parameters that were used together in parameter mapping appear to have intertwined effects that may be difficult to predict, thus requiring a trial-and-error strategy to improve the sonification effectiveness.

In our research, volatiles were not tested for their hedonic perception (i.e., as pleasant versus unpleasant), but detectability and repellent properties. Nevertheless, a next step in the sonification approach of volatiles may be to start with floral bouquets. The audio should then be tested on humans in an adapted experimental setup. An example might be testing human panellists for a sensory evaluation by which they rate the audio (un)pleasantness. Such a setup would also include a combination of sound parameters other than Lpeak to quantify the pleasantness of odour perception via sound traits such as pitch [61] or rhythm [15]. This sonification approach needs to be phased in, distinguishing between chemical attributes of single volatiles and relative concentrations in mixtures of volatiles. Still another application of the sonification described here would be to test feeding deterrent compounds, acting on gustation, that are probably even more diversified in nature than volatiles.

## 5. Conclusions

Sonification is a heuristic technique that presents new perspectives in data representation, interpretation, and understanding. Here, we showed that parameter mapping between chemical attributes of volatiles and sound parameters leads to estimating the level of olfactory perception of those chemicals by humans. Sonification is constrained, as are other modelling systems [35,39,40,43,44,45,46], by the specific settings involved in its implementation. In this context, the stepwise enhancement of mapping conditions by the setting of randomized chemical–sound nodes resulted gradually in sounds that more reliably reflect the volatile bioactivity. However, we are aware that the bivariate plot graph included more than one outlier, and that the strength of the correlations between the sound trait Lpeak with the human olfactory threshold remained relatively weak. Whether the chemical–sound correspondence can be further enhanced by omitting some chemical attributes and/or by adding new ones, thus also adding new sound parameters, requires further investigation.

## Figures and Tables

**Table 1:** Mapping conditions and statistical results obtained by comparing human olfactory thresholds with gathered peak sound pressures. Each mapping condition with a reference code (set) was applied to 100 molecules by running the Processing application (Figure 2). Part of the mapping nodes was determined randomly (mapping goal). Lpeak values were calculated from the resulting audio. These values were then statistically compared with the SHOT d1 values, using the Spearman rank-order correlations with *p* values (*p*), two-tailed, at α = 0.05. Sample size (n); Spearman’s correlation coefficient (r_S_); size of difference relative to variation in the sample data (t). The *p* values remaining statistically significant after Holm’s sequential Bonferroni correction are given in bold. For more explanation, see text.

Set	Mapping Condition	Mapping Goal	r_S_	t	*p*
set01	A2-D1--C3-E7-F4-G5-H8-I6-J9	random C E F G H I J	0.1529	1.53	0.129
set02	A2-D1--C5-E3-F6-G7-H9-I4-J8	random C E F G H I J	0.465	5.2	**<0.001**
set03	A2-D1--C6-E3-F8-G4-H7-I5-J9	random C E F G H I J	0.4738	5.33	**<0.001**
set04	A2-D1--C4-E9-F6-G5-H3-I7-J8	random C E F G H I J	0.3194	3.34	**0.001**
set05	A2-D1--C4-E5-F8-G6-H7-I9-J3	random C E F G H I J	0.2514	2.57	0.012
set06	A2-D1--C8-E3-F6-G7-H9-I4-J5	random C E F G H I J	0.3572	3.79	**<0.001**
set07	A2-D1--C6-E3-F4-G7-H5-I8-J9	random C E F G H I J	0.46	5.13	**<0.001**
set08	A2-D1--C5-E6-F3-G7-H9-I8-J4	random C E F G H I J	0.2048	2.07	0.041
set09	A2-D1--C7-E3-F4-G9-H8-I5-J6	random C E F G H I J	0.5212	6.04	**<0.001**
set10	A2-D1--C3-E7-F6-G4-H5-I8-J9	random C E F G H I J	0.0917	0.91	0.365
set11	A2-D1--C9-E3-F8-G7-H6-I5-J4	random C E F G H I J	0.2718	2.8	0.006
set12	A2-D1--C7-E8-F5-G3-H4-I6-J9	random C E F G H I J	0.3874	4.16	**<0.001**
set13	A2-D1--C4-E7-F3-G8-H9-I5-J6	random C E F G H I J	0.2424	2.47	0.015
set14	A2-D1--C4-E3-F5-G8-H6-I7-J9	random C E F G H I J	0.4246	4.64	**<0.001**
set15	A2-D1--C3-E7-F8-G4-H9-I5-J6	random C E F G H I J	−0.0325	−0.32	0.750
set16	A2-D1--C9-E4-F3-G5-H6-I8-J7	random C E F G H I J	0.2613	2.68	0.009
set17	A2-D1--C8-E7-F9-G3-H4-I5-J6	random C E F G H I J	0.186	1.87	0.064
set18	A2-D1--C5-E9-F4-G6-H7-I8-J3	random C E F G H I J	0.3953	4.26	**<0.001**
set19	A2-D1--C7-E3-F9-G6-H4-I5-J8	random C E F G H I J	0.2825	2.92	**0.004**
set20	A2-D1--C9-E4-F3-G5-H6-I7-J8	random C E F G H I J	0.3283	3.44	**0.001**
set21	A2-D1--C3-E7-F4-G6-H5-I8-J9	random C E F G H I J	0.2306	2.35	0.021
set22	A2-D1--C6-E4-F8-G7-H9-I5-J3	random C E F G H I J	0.4456	4.93	**<0.001**
set23	A2-D1--C5-E3-F8-G6-H7-I9-J4	random C E F G H I J	0.4013	4.34	**<0.001**
set24	A2-D1--C9-E3-F4-G6-H5-I8-J7	random C E F G H I J	0.5892	7.22	**<0.001**
set26	A2-D1--C9-E3-F4-G7-H5-I8-J6	random C G H I J	0.5789	7.03	**<0.001**
set27	A2-D1--C5-E3-F4-G7-H8-I6-J9	random C G H I J	0.6049	7.52	**<0.001**
set28	A2-D1--C6-E3-F4-G5-H7-I8-J9	random C G H I J	0.5681	6.83	**<0.001**
set29	A2-D1--C5-E3-F4-G7-H9-I6-J8	random C G H I J	0.663	8.77	**<0.001**
set30	A2-D1--C5-E3-F4-G9-H6-I8-J7	random C G H I J	0.5095	5.86	**<0.001**
set54	A2-D1--C9-E3-F4-G7-H6-I5-J8	random C G H I J	0.5951	7.33	**<0.001**
set55	A2-D1--C7-E3-F4-G5-H6-I8-J9	random C G H I J	0.5711	6.89	**<0.001**
set56	A2-D1--C9-E3-F4-G6-H7-I8-J5	random C G H I J	0.6461	8.38	**<0.001**
set57	A2-D1--C8-E3-F4-G6-H7-I5-J9	random C G H I J	0.7138	10.09	**<0.001**
set58	A2-D1--C7-E3-F4-G8-H9-I5-J6	random C G H I J	0.5539	6.59	**<0.001**
set59	A2-D1--C6-E3-F4-G8-H7-I9-J5	random C G H I J	0.4435	4.9	**<0.001**
set60	A2-D1--C5-E3-F4-G6-H7-I8-J9	random C G H I J	0.6306	8.04	**<0.001**
set61	A2-D1--C6-E3-F4-G5-H7-I9-J8	random C G H I J	0.5282	6.16	**<0.001**
set62	A2-D1--C5-E3-F4-G8-H9-I7-J6	random C G H I J	0.5202	6.03	**<0.001**
set63	A2-D1--C7-E3-F4-G8-H6-I9-J5	random C G H I J	0.4822	5.45	**<0.001**
set64	A2-D1--C9-E3-F4-G7-H5-I6-J8	random C G H I J	0.648	8.42	**<0.001**
set65	A2-D1--C5-E3-F4-G9-H6-I7-J8	random C G H I J	0.5155	5.96	**<0.001**
set66	A2-D1--C5-E3-F4-G6-H7-I9-J8	random C G H I J	0.6062	7.55	**<0.001**
set67	A2-D1--C5-E3-F4-G7-H6-I9-J8	random C G H I J	0.4492	4.98	**<0.001**
set68	A2-D1--C6-E3-F4-G5-H9-I7-J8	random C G H I J	0.5061	5.81	**<0.001**
set69	A2-D1--C9-E3-F4-G6-H5-I7-J8	random C G H I J	0.6192	7.81	**<0.001**
set70	A2-D1--C6-E3-F4-G8-H9-I7-J5	random C G H I J	0.3521	3.72	**<0.001**
set71	A2-D1--C8-E3-F4-G5-H7-I6-J9	random C G H I J	0.7172	10.19	**<0.001**
set72	A2-D1--C7-E3-F4-G5-H8-I9-J6	random C G H I J	0.502	5.75	**<0.001**
set57b	A2-D1--C8-E3-F4-G6-H7-I5-J9	set57 but Feedback 0–64	0.6479	8.42	**<0.001**
set73	A2-D1--C8-E3-F4-G5-H7-I6-J9	set71 but Feedback 0–64	0.5136	5.93	**<0.001**

**Table 2:** Correlations between bioactivity datasets. Results from Spearman rank-order correlations with *p* values (*p*), two-tailed, at α = 0.05. Sample size (n); Spearman’s correlation coefficient (r_S_); size of difference relative to variation in the sample data (t); and degrees of freedom (df). The three *p* values remained significant after Holm’s sequential Bonferroni correction. The variables refer to Table S1. The second listed comparison, SHOT d1 vs. Lpeak, refers to ‘set57b’ in Table 1 and is illustrated in Figure 3.

Variable Comparison	n	r_S_	t	df	*p*
SHOT d1 vs. SHOT d2	272	0.9858	96.62	270	5 × 10^−7^
SHOT d1 vs. Lpeak	100	0.6479	8.42	98	<1 × 10^−6^
Log(1/ODT) vs. Lpeak	100	0.6837	9.27	98	<1 × 10^−6^

## Data Availability

The Massive preset sound used in this study has a proprietary format. This preset sound and the audio clips produced in this study are available on request from the corresponding author. Similar audios are available as MP3 files from Freesound: https://freesound.org/people/jlboeve/packs/30377/ (accessed on 14 February 2023). The two Processing scripts are published [12].

## Supplementary Materials

The following supporting information can be downloaded at: https://www.mdpi.com/article/10.3390/biology12050670/s1, Table S1: Descriptors and bioactivities of the molecules used in the study; Table S2: Statistical results from multiple linear regressions.
